# Meniscal Ramp Injury Diagnosis

**DOI:** 10.1055/s-0044-1791791

**Published:** 2024-12-07

**Authors:** Pedro Baches Jorge, Diego Escudeiro de Oliveira, Guilherme do Amaral Mussatto, Melanie Mayumi Horita, Victor Eduardo Roman Salas, Rafael Baches Jorge

**Affiliations:** 1 Irmandade da Santa Casa de Misericórdia de São Paulo, São Paulo, SP, Brasil

**Keywords:** anterior cruciate ligament injuries, arthroscopy, tibial meniscus injuries

## Abstract

**Objective** : This study compared diagnostic methods for meniscal ramp injury (magnetic resonance imaging [MRI], arthrotomography, and arthroscopy) to determine the most sensitive and the agreement level between them.

**Method:** We studied 21 patients, all young athletes with suspected anterior cruciate ligament (ACL) injury after trauma for at least 3 months and no evidence or history of other osteoarticular injuries in the knee. The patients underwent MRI and arthrotomography. Following ACL injury confirmation, they underwent arthroscopy for ligament reconstruction and evaluation of the medial meniscus to confirm or exclude a ramp injury. McNemar's agreement test compared the diagnostic methods. We also assessed specificity and sensitivity using arthroscopy as the gold standard with a 95% confidence interval and p < 0.005.

**Result** : The results were consistent with the literature. MRI had 73.3% sensitivity and 83.3% specificity, with 76.2% agreement with the gold standard. Arthrotomography sensitivity and specificity were 100% and 66.7%, respectively, with 90.5% agreement with arthroscopy.

**Conclusion** : In our study, arthrotomography was the most sensitive diagnostic method and had the highest agreement with the gold standard. We recommend its consideration for diagnosing ACL injuries.

## Introduction

**Fig. 1:**
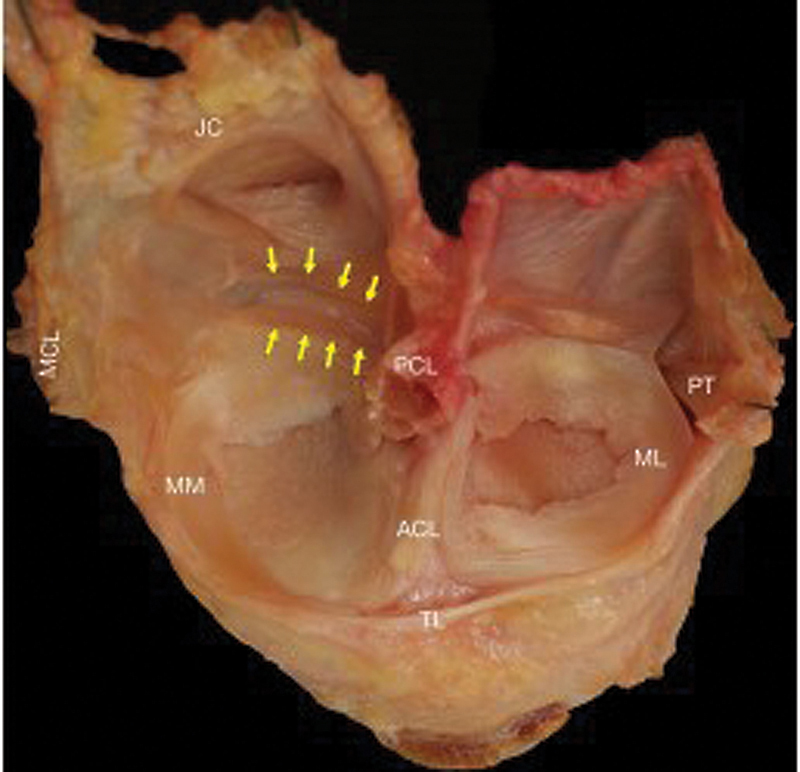
Anatomical dissection of the left knee joint. Yellow arrows mark the posteromedial femoral recess. Medial collateral ligament (MCL), medial meniscus (MM), lateral meniscus (ML), joint capsule (JC), posterior cruciate ligament (PCL), anterior cruciate ligament (ACL), and enlargement of the posteromedial femoral recess. Source: Śmigielski R, Becker R, Zdanowicz U, Ciszek B. Medial meniscus anatomy from basic science to treatment. Knee Surg Sports Traumatol Arthrosc 2015;23(1): 8-14.

Hamberg et al. described the meniscal ramp injury in the 1980s as a lesion of the posterior aspect of the medial meniscus in the topography shown by Fig. 1 , and, later, Strober apud Sonnery-Cottet et al. 1 described it as a longitudinal lesion, 2.5 cm long, at the meniscocapsular junction. These lesions are more common in male patients with anterior cruciate ligament (ACL) rupture and a mean age of 30 years old. The classification of this type of injury relies on the affected area: type 1 includes meniscocapsular injuries in the outermost region of the synovial sheath; type 2 consists of upper partial injuries, which are stable and only diagnosed at a transoperative approach; type 3 includes lower partial or occult injuries, not visible at a transoperative approach; type 4 consists of complete injuries in the red-red zone; and type 5 includes double ruptures involving the meniscocapsular junction and the anterior portion of the posterior horn 2 ( Fig. 1 ) .

The literature has no consensus regarding the most sensitive and specific method for diagnosing a meniscal ramp injury. 3 One method is the intraoperative detection of the injury during arthroscopy for ACL repair due to clinical suspicion, specific physical examination tests (pivot shift grade III and Lachman grade III), and suggestive epidemiology, such as male gender and young age (30 years old). 4 Another possibility is magnetic resonance imaging (MRI) showing an injury with hyperintense signal on T2-weighted images in the posterior topography of the medial meniscus at the meniscocapsular junction and edema of the posterior medial tibial plateau. 5 A third diagnostic method is arthrotomography with intra-articular contrast injection; its sensitivity is consistent with MRI per the current literature. After diagnosis, treatment of these lesions can be conservative or surgical. Surgical repair is the most common treatment, with good clinical outcomes and a low failure rate. 6 Thaunat et al. 2 stated that a ramp lesion repair can occur through anterior arthroscopic portals and all-inside sutures. However, creating a posteromedial accessory portal facilitates debridement for lesion access, allows better visualization of its complete repair, and helps the positioning of vertical sutures in the deep meniscal fibers, with a biomechanical advantage over sutures Sonnery-Cottet et al. 7 added that this additional portal aimed at better visualization of the posterior region of the medial meniscus, helping to discover a percentage of lesions that could go undiagnosed through a standard anterior exploration. Using this technique, arthroscopic repair of meniscal ramp injuries during ACL reconstruction provided a high healing rate, making it a reliable surgical procedure for treating this type of lesion

Using this technique, arthroscopic repair of meniscal ramp injuries during ACL reconstruction provided a high healing rate, making it a reliable surgical procedure for treating this type of lesion. 3 8 Underdiagnosis and failure to treat these injuries can directly influence rehabilitation after ACL reconstruction and culminate in anteroposterior and rotatory knee instability. 3 9

## Materials and methods

The ethics committee of our institution approved this study under number CAAE 46245121.5.0000.5479.

We studied 21 patients, all young athletes with suspected ACL injury after trauma at least 3 months previously and no evidence or history of other osteoarticular injuries in the knee. We excluded patients with current infection in the injured knee, chronic disease, severe conditions preventing surgical intervention, or previous knee injury. The study lasted for 2 years. Five radiologists assessed the selected patients, who underwent MRI, arthrotomography, and arthroscopy. Initially, an MRI of the affected knee occurred on a 1.5-T device. Immediately after, we performed an arthrotomography on a 64-channel device with an intra-articular injection of 15 mL of iopromide.

After imaging tests confirmed the ligament injury, the patients underwent an arthroscopic surgical procedure for ACL reconstruction. Knee-specialized surgeons performed the procedure, starting with anterolateral and anteromedial portals to assess the medial meniscus and the presence or absence of a ramp lesion. Next, they did an all-inside suturing in positive cases. If there was diagnostic doubt or non-diagnosis of the lesion through anterior portals, they created a posteromedial portal to confirm its existence and proceeded with the ACL reconstruction.

Table 1 compares the diagnostic methods per the association between the presence or absence of a meniscal ramp lesion. McNemar's agreement test compared arthrotomography and MRI. We calculated specificity and sensitivity using arthroscopy as the gold standard with a 95% confidence interval and p < 0.05.

**Table 1:** Comparison between arthrotomography, magnetic resonance imaging, and arthroscopy per the positivity of the diagnostic test

Diagnostic method	Number of patients
**Arthrotomography**	
Positive	17
Negative	4
**Magnetic resonance imaging**	
Positive	12
Negative	9
**Arthroscopy**	
Positive	15
Negative	6

## Results

Twelve patients (57%) with a mean age of 26, nine men and three women, had a positive result for meniscal ramp lesion in MRI. Meanwhile, nine patients (43%) with a mean age of 23, four men and five women, had a negative result. In the arthrotomography study, 17 patients (81%) with a mean age of 26, 11 men and six women, had a positive result for the lesion, and four patients (19%) with a mean age of 19, two men and two women, had a negative result. However, in the arthroscopic study, 15 patients (71%) with a mean age of 26, 11 men and four women, had a positive result, and six patients (29%) with a mean age of 19, two men and four women, had a negative result ( Table 1 ). There was agreement for test positivity between the imaging methods and the arthroscopic study in 15 patients (71%). Furthermore, the lesion was positive in an arthroscopic procedure after a positive arthrotomography test and a negative MRI in four patients (19%). All patients underwent surgery within 3 months of the trauma ( Table 1 ).

The agreement between arthrotomography and arthroscopy was 90.5% (p = 0.5) using the McNemar test. Agreement between MRI and arthroscopy was 76.2% (p = 0.375). Arthrotomography had 100% sensitivity (lower limit = 90.4%, upper limit = 100.0%) and 66.7% specificity (lower limit = 5.8%, upper limit = 100.0%) with 95% CI. MRI showed 73.3% sensitivity (lower limit = 50.2%, upper limit = 100.0%) and 83.3% specificity (lower limit = 35.1%, upper limit =100.0%), also with 95% CI ( Table 2 ).

**Table 2:** Statistical analysis using the McNemar's method

Test type			Arthroscopy		Total
			Positive	Negative	
Arthrotomography	Positive	N	15	2	17
		%	71.4%	9.5%	81.0%
	Negative	N	0	4	4
		%	0.00%	19.0%	19.0%
Total		N	15	6	21
		%	71.40%	28.60%	100.00%
McNemar's test for agreement (p = 0.500)					
agreement	90.5%				
disagreement	9.5%				

**Table:** 

			Arthroscopy		Total
			Positive	Positive	
Magnetic resonance imaging	Positive	N	11	1	12
		%	52.4%	4.8%	57.1%
	Negative	N	4	5	9
		%	19.0%	23.8%	42.9%
Total		N	15	6	21
		%	71.4%	28.6%	100.0%
McNemar's test for agreement (p = 0.375)					
agreement	76.2%				
disagreement	23.8%				

**Table:** 

	Value	95% CI	
		Lower limit	Upper limit
Sensitivity	100.0%	90.4%	100.0%
Specificity	66.7%	5.8%	100.0%
PPV	88.2%	68.9%	100.0%
NPV	100.0%	70.5%	100.0%

**Table:** 

	Value	95% CI	
		Lower limit	Upper limit
Sensitivity	73.3%	50.2%	100.0%
Specificity	83.3%	35.1%	100.0%
PPV	91.7%	71.0%	100.0%
NPV	55.6%	10.0%	100.0%

Abbreviations: CI, Confidence interval; NPV, negative predictive value; PPV, positive predictive value.

## Discussion

Liu et al. 10 described the ramp lesion as a singular type of medial meniscal injury with a high association with ACL rupture. Its prevalence increases over time since the ACL injury. Therefore, this type of injury requires early diagnosis.

Regarding diagnostic methods, intraoperative detection of meniscal injury during arthroscopy for ACL is the gold standard for investigation. 4 Chahla et al. 11 also stated that arthroscopic evaluation is essential to detect meniscal ramp injuries. Sonnery-Cottet et al. 9 described that a systematic exploration of the posteromedial compartment of the knee is mandatory to identify ramp lesions.

Following diagnosis, treatment of these injuries can be conservative. Shelbourne and Rask 12 evaluated the need for suture repair versus treatment with follow-up and injury ablation based on the stability or instability of the meniscal tear (i.e., displaceable to the intercondylar notch upon stress), concluding that stable injuries even greater than 1 cm in length can undergo ablation and remain asymptomatic and with no destabilization. Arthroscopic repair, especially in unstable injuries, was the most widely used approach, with good clinical outcomes and a low failure rate .6 Arthroscopic repair of meniscal ramp injuries during ACL reconstruction provided a high healing rate and is a reliable surgical procedure to treat these injuries. 8 Hatayama et al. 3 studied 57 patients undergoing ACL reconstruction and presenting meniscal ramp injuries, opting for repair in 25 patients. They demonstrated that the meniscal healing rate was significantly higher in these patients and that this fact directly implies the anterior knee laxity and avoids increased forces on the graft.

According to recent research, MRI sensitivity ranges from 48% to 84.6%. 3 4 13 These values are consistent with our study, with a 73.3% sensitivity. Moreover, MRI had a lower agreement (76.2%) than the gold standard diagnostic method, i.e., arthroscopy, compared with arthrotomography (90.5%).

Chahla et al. 11 found that intra-articular contrast infusion can help detect lesions between the meniscus and the capsule that may not be visible on MRI. These authors found a sensitivity ranging from 84% to 100% for arthrotomography detecting these lesions. In our study, arthrotomography had a higher sensitivity of 100%.

Similarly, Greif et al. 14 demonstrated high sensitivity and specificity for the contrast test, but their study warns about the lack of consistent data in the literature comparing its effectiveness with conventional MRI.

Our results indicate that arthrotomography is the test most resembling the gold standard, warranting its consideration in cases with suspected ramp injury associated with ACL injury. Nevertheless, unlike MRI, arthrotomography involves the injection of intra-articular contrast. Therefore, it is an invasive examination with risks, and it is critical to select correctly patients benefiting from it. In addition, arthrotomography is not available in all services, making its access to certain patients difficult. As such, due to the small sample size, there was no statistical significance for sensitivity and specificity because of the wide range between lower and upper limits.

Our study reiterates the need for a larger sample to reliably evaluate the best diagnostic method for medial meniscal ramp lesions.

## Conclusion

In our study, arthrotomography presented results closer to the gold standard, i.e., arthroscopy, with high sensitivity. MRI results were consistent with the literature, being less sensitive than arthrotomography. However, one must consider factors directly related to the performance of these imaging methods. More reliable results require further studies with a larger sample of patients.
