# Supplementary material for: Meniscal Ramp Injury Diagnosis
Source: Rev Bras Ortop (Sao Paulo). 2024 Dec 7;59(5):e702–6. doi: 10.1055/s-0044-1791791 (PMC11624941; doi:10.1055/s-0044-1791791)
Supplement: Related texts

# Research article: Diagnóstico das lesões meniscais em rampa

**Keywords:** artroscopia, lesões do ligamento cruzado anterior, lesões do menisco tibial

## Abstract

**Objetivo** : Comparar os métodos diagnósticos para lesão meniscal em rampa (ressonância magnética, artrotomografia e artroscopia) evidenciando qual o método mais sensível e qual o grau de concordância entre os métodos.

**Método:** Foram estudados 21 pacientes, sendo esses, jovens, atletas, com suspeita de lesão do LCA após trauma há pelo menos 3 meses, sem evidências ou histórico de outras lesoes osteoarticulares no joelho. Para isso os pacientes foram submetidos a ressonância magnética, artrotomografia e após a confirmação da lesão do LCA foi realizada artroscopia para reconstrução ligamentar e avaliação do menisco medial para confirmar ou excluir o diagnóstico de lesão em rampa. Os métodos diagnósticos foram comparados pelo teste de concordância de McNemar, sendo também avaliada especificidade e sensibilidade, utilizando como padrão ouro a artroscopia, com intervalo de confiança de 95% e p < 0,005.

**Resultado** : Os resultados foram semelhantes com os já encontrados na literatura. A ressonância magnética apresentou sensibilidade de 73,3% e especificidade de 83,3%, com uma concordância de 76,2% com o padrão ouro. Já a artrotomografia apresentou sensibilidade e especificidade de 100% e 66,7% respectivamente, com uma concordância de 90,5% com a artroscopia.

**Conclusão** : Neste trabalho, a artrotomografia se apresentou como o método diagnóstico mais sensível e com maior concordância com o padrão ouro, devendo ser considerada na investigação diagnóstica de pacientes com lesão do LCA.

## Introdução

A lesão meniscal em rampa foi descrita na década de 1980 por Hamberg et al. como uma lesão do aspecto posterior do menisco medial na topografia exemplificada na Fig. 1 e em seguida descrita por Strober apud Sonnery-Cottet et al. 1 como uma lesão longitudinal, com 2,5 cm de comprimento, localizado na junção meniscocapsular. Essas lesões estão mais presentes em pacientes com ruptura do ligamento cruzado anterior (LCA), do sexo masculino e em média de 30 anos de idade. Esse tipo de lesão é classificado de acordo com a zona de acometimento: tipo 1 - lesões meniscocapsulares, essas lesões estão localizadas muito perifericamente na bainha sinovial - tipo 2 - lesões parciais superiores, essas lesões são estáveis e podem ser diagnosticadas apenas por uma abordagem trans-operatória - tipo 3 - lesões parciais inferiores ou ocultas, essas lesões não são visíveis com a abordagem trans-operatória - tipo 4 - lesão completa na zona vermelha-vermelha - tipo 5 - dupla ruptura envolvendo a junção meniscocapsular e a porção anterior do corno posterior 2 ( Fig. 1 ) .

**Fig. 1:** Dissecção anatômica da articulação do joelho esquerdo. O recesso femoral póstero-medial é marcado com setas amarelas. Ligamento colateral medial (MCL), menisco medial (MM), menisco lateral (ML) cápsula articular (JC), ligamento cruzado posterior (PCL), ligamento cruzado anterior (ACL), ampliação do recesso femoral póstero-medial. Fonte: Śmigielski R, Becker R, Zdanowicz U, Ciszek B. Medial meniscus anatomy from basic science to treatment. Knee Surg Sports Traumatol Arthrosc 2015;23(1): 8-14.

Ainda não há consenso na literatura a respeito do método mais sensível e específico para diagnosticar uma lesão meniscal em rampa. 3 Um dos métodos utilizados é a detecção intraoperatória da lesão, no momento da artroscopia para reparo do ligamento cruzado anterior através de suspeita clínica prévia com testes especiais de exame físico (Pivot Shift grau III e Lachman grau III) e epidemiologia sugestiva como sexo masculino e paciente jovem (30 anos). 4 Outra possibilidade, é a ressonância magnética que, na presença de lesão, apresenta hipersinal na imagem ponderada em T2 na topografia posterior do menisco medial na junção meniscocapsular, além de edema do platô tibial medial posterior. 5 Esse método diagnóstico apresenta baixa sensibilidade e com risco de resultado falso negativo quando realizado com o joelho em extensão em quadros de lesão aguda. Outro método diagnóstico é a artrotomografia, na qual é realizada injeção de contraste intraarticular, com sensibilidade semelhante ao exame de ressonância magnética, segundo a literatura atual. Uma vez diagnosticada, essas lesões podem ser tratadas de forma conservadora ou a partir do reparo cirúrgico da lesão, sendo esta a abordagem mais utilizada, com bons resultados clínicos e baixa taxa de falhas. 6 O reparo da lesão em rampa de acordo com Thaunat et al., 2 pode ser feito através de portais artroscópicos anteriores e sutura all-inside , porêm a realização de um portal acessório posteromedial facilita o desbridamento para acesso da lesão, melhor visualização de reparo completo da mesma alêm de facilitar o posicionamento de suturas verticais as fibras profundas dos meniscos trazendo vantage biomecanica à sutura . Este portal adicional de acordo com Sonnery-Cottet et al., 7 devido sua melhor capacidade de visualização da região posterior dos menisco medial, pode ajudar na descoberta de uma porcentagem de lesões que poderiam ser não diagnosticadas através de uma exploração anterior padrão.

Através dessa técnica o reparo meniscal artroscópico das lesões em rampa durante a reconstrução do LCA proporcionou uma alta taxa de cicatrização, sendo um procedimento cirúrgico confiável para o tratamento deste tipo de lesão. 3 8 O subdiagnóstico e o não tratamento dessas lesões podem influenciar diretamente na reabilitação pós reconstrução do LCA e culminar em instabilidades ântero-posterior e rotatória do joelho. 3 9

## Materiais e métodos

O referido trabalho teve a aprovação do comitê de ética da nossa instiutição sob o número CAAE- 46245121.5.0000.5479.

Foram estudados 21 pacientes, sendo esses, jovens, atletas, com suspeita de lesão do LCA após trauma há pelo menos 3 meses, sem evidências ou histórico de outras lesões osteoarticulares no joelho. Foram excluídos pacientes com infecção atual no joelho lesionado, doença crônica, doença grave que impedisse a abordagem cirúrgica ou lesão prévia no joelho. O estudo foi realizado por um período de 2 anos. Os pacientes selecionados foram estudados por 5 radiologistas sendo submetidos a ressonância magnética, artrotomografia e artroscopia. Inicialmente, foi realizada ressonância magnética do joelho acometido em aparelho de 1.5 T. Logo após, foi feita artrotomografia em aparelho de 64 canais, com injeção de 15 ml de iopromida intraarticular.

Após a realização dos exames de imagem e confirmação da lesão ligamentar, foi realizado procedimento cirúrgico de artroscopia para reconstrução do LCA. O procedimento foi realizado por cirurgiões especializados em joelho, iniciando através de portais antero-lateral e antero-medial sendo realizada avaliação do menisco medial e a presença ou não de lesão em rampa, seguido de sutura all-inside do mesmo em casos positivos, em caso de dúvida diagnóstica ou não diagnóstico da lesão por portais anteriores foi realizado portal posteromedial para confirmação da existência da mesma, em seguida foi realizada a reconstrução do LCA

Os métodos diagnósticos foram comparados através de uma associação entre a presença ou não de lesão meniscal em rampa, conforme ( Tabela 1 ). Foi realizado teste de concordância através do método de McNemar para comparar a artrotomografia e a ressonância magnética, sendo também calculadas a especificidade e a sensibilidade utilizando como padrão ouro a artroscopia, com intervalo de confiança de 95% e p < 0,05.

**Tabela 1:** Comparativo entre artrotomografia, ressonância magnética e artroscopia através da positividade do exame diagnóstico

Método diagnóstico	N° de pacientes
**Artrotomografia**	
Positivo	17
Negativo	4
**Ressonância magnética**	
Positivo	12
Negativo	9
**Artrocopia**	
Positivo	15
Negativo	6

## Resultados

Foram encontrados 12 pacientes (57%) com uma média de 26 anos sendo 9 homens e 3 mulheres com resultado positivo para lesão meniscal em rampa no estudo de ressonância magnética e 9 pacientes (43%) com uma média de 23 anos sendo 4 homens e 5 mulheres com resultado negativo. No estudo de artrotomografia foram identificados 17 pacientes (81%) com uma média de 26 anos sendo 11 homens e 6 mulheres com resultado positivo para lesão e 4 pacientes (19%) com uma média de 19 anos sendo 2 homens e 2 mulheres com resultado negativo. No entanto, no estudo artroscópico foram encontrados 15 pacientes (71%) com uma média de 26 anos sendo 11 homens e 4 mulheres com resultado positivo para a mesma lesão e 6 pacientes (29%) com uma média de 19 anos sendo 2 homens e 4 mulheres com resultado negativo ( Tabela 1 ). Houve concordância para positividade do teste entre os métodos de imagem e estudo artroscópico em 15 pacientes (71%). Além disso, foi confirmada positividade da lesão em procedimento artroscópico após teste positivo para artrotomografia e negativo na ressonância magnética em 4 pacientes (19%). Todos os pacientes foram operados em até 3 meses do trauma ( Tabela 1 ).

A concordância entre artrotomografia e artroscopia foi de 90,5% (p = 0,5) utilizando o teste de McNemar. Já entre ressonância magnética e artroscopia foi de 76,2% (p = 0,375). A sensibilidade da artrotomografia foi de 100% (LI= 90,4%,LS = 100,0%) e especificidade de 66,7% (LI= 5,8%,LS = 100,0%) com IC 95%. A ressonância magnética apresentou sensibilidade de 73,3% (LI= 50,2%,LS = 100,0%) e especificidade de 83,3% (LI= 35,1%,LS = 100,0%) também com IC 95%, conforme ( Tabela 2 ).

**Tabela 2:** Análise estatística através do método de McNemar

Tipo de exame			Artroscopia		Total
			Positivo	Negativo	
Artrotomografia	Positivo	N	15	2	17
		%	71.4%	9.5%	81.0%
	Negativo	N	0	4	4
		%	0.00%	19.0%	19.0%
Total		N	15	6	21
		%	71.40%	28.60%	100.00%
Teste de concordância de McNemar (p = 0,500)					
concordância	90.5%				
discordância	9.5%				

**Table:** 

			Artroscopia		Total
			Positivo	Negativo	
Ressonância	Positivo	N	11	1	12
		%	52.4%	4.8%	57.1%
	Negativo	N	4	5	9
		%	19.0%	23.8%	42.9%
Total		N	15	6	21
		%	71.4%	28.6%	100.0%
Teste de concordância de McNemar (p = 0,375)					
concordância	76.2%				
discordância	23.8%				

**Table:** 

	Valor	IC 95%	
		LI	LS
Sensibilidade	100.0%	90.4%	100.0%
Especificidade	66.7%	5.8%	100.0%
VPP	88.2%	68.9%	100.0%
VPN	100.0%	70.5%	100.0%

**Table:** 

	Valor	IC 95%	
		LI	LS
Sensibilidade	73.3%	50.2%	100.0%
Especificidade	83.3%	35.1%	100.0%
VPP	91.7%	71.0%	100.0%
VPN	55.6%	10.0%	100.0%

Abbreviations: IC, intervalo de confiança; VPN, valor preditivo negative; VPP, valor preditivo positivo.

## Discussão

A lesão em rampa é descrita por Liu et al. 10 como um tipo especial de lesão do menisco medial com alta prevalência associada à ruptura do ligamento cruzado anterior. Sua prevalência aumenta com o tempo da lesão do LCA. Dessa forma se faz necessário o diagnóstico precoce desse tipo de lesão.

Com relação aos métodos diagnósticos, a detecção intraoperatória da lesão meniscal, na artroscopia para reparo do ligamento cruzado anterior é o método padrão-ouro de investigação. 4 Chahla et al. 11 tambem afirma que para detecção de lesão meniscal em rampa é imprescindível uma avaliação artroscópica. Sonnery-Cottet et al. 9 descrevem que uma exploração sistemática do compartimento póstero-medial do joelho é obrigatória para identificar com segurança lesões na rampa.

Uma vez diagnosticadas, essas lesões podem ser tratadas conservadoramente, Shelbourne e Rask 12 avaliaram a necessidade de reparo de sutura versus tratamento com acompanhamento e ablação da lesão com base em se a ruptura do menisco é estável ou instável (deslocável para incisura intercondilar ao stress) e concluíram que lesões estáveis, mesmo maiores que 1 cm de comprimento, podem ser tratadas com ablação permanecendo assintomática e sem instabilização ou com reparo artroscópico, principalmente em lesões instáveis, sendo a abordagem mais utilizada, com bons resultados clínicos e baixa taxa de falha. 6 O reparo meniscal artroscópico das lesões em rampa durante a reconstrução do LCA proporcionou uma alta taxa de cicatrização, sendo um procedimento cirúrgico confiável para o tratamento dessas lesões. 8 Hatayama et al. 3 estudaram um total de 57 pacientes submetidos a reconstrução do LCA e presença de lesão meniscal em rampa, sendo optado por reparo em 25 pacientes, foi demonstrado que a taxa de cicatrização meniscal foi significativamente maior nesses pacientes e que esse fato implica diretamente na frouxidão anterior do joelho e evita aumento de forças sobre o enxerto.

A ressonância magnética apresenta uma sensibilidade que varia de 48% a 84,6% de acordo com estudos recentes, 3 4 13 acontecendo de forma semelhante no atual estudo, sendo encontrado uma sensibilidade de (73,3%), alêm do que a ressonância magnética apresentou-se com uma concordância menor (76,2%) em relação ao metodo diagnóstico padrão ouro - artroscopia de forma comparativa com a artrotomografia (90,5%).

Chahla et al. 11 constataram que a infusão de contraste intra-articular pode ajudar na detecção de lesões que se localizam entre o menisco e a cápsula e que podem não ser visíveis no exame de ressonância magnética, o autor encontrou uma sensibilidade de 84-100% para a artrotomografia na detecção dessas lesões. Sendo encontrado sensibilidade ainda mais expressiva no presente estudo em que a artrotomografia apresentou 100% de sensibilidade ao método.

Do mesmo modo, Greif et al. 14 demonstraram alta sensibilidade e especificidade do exame contrastado. No entanto, o estudo alerta para a ausência de dados consistentes na literatura comparando sua eficácia com a ressonância magnética convencional.

Os resultados deste estudo apontam para artrotomografia como o exame que mais se assemelha ao padrão ouro, devendo ser considerado nos casos com suspeita de lesão em rampa associada à lesão do LCA. No entanto, diferente da ressonância magnética, a artrotomografia envolve a injeção de contraste intraarticular, sendo um exame invasivo e não isento de riscos, tornando importante a seleção correta dos pacientes que irão se beneficiar do exame. Além disso, a artrotomografia não é um exame disponível em todos os serviços dificultando o acesso a determinados pacientes.

Apesar dos dados encontrados neste estudo serem semelhantes aos dos estudos já realizados, existe alguns viéses encontrados devido a avaliação realizada por meio dos métodos de imagem propostos poderem apresentar falsos negativos em traumas agudos com exame realizado com o joelho em extensão e o tamanho amostral de pacientes não ser expressivo, não havendo significância estatística visto que na avaliação de sensibilidade e especificidade houve intervalo amplo entre o limite inferior e superior, o que pode ser explicado por esse tamanho reduzido da amostra.

Logo, o estudo reitera a necessidade de uma maior amostra para avaliar de forma fidedigna o melhor método diagnóstico para lesão em rampa do menisco medial

## Conclusão

Neste trabalho, a artrotomografia apresentou resultado mais próximo do padrão ouro - artroscopia, com uma alta sensibilidade. A ressonância magnética mostrou resultados semelhantes aos já encontrados na literatura, sendo menos sensível que o estudo por artrotomografia. No entanto, fatores diretamente relacionados à realização dos exames de imagem descritos devem ser considerados e ainda são necessários mais estudos com uma amostra maior de pacientes para um resultado mais fidedigno.
